# Complete plastid genome sequences suggest strong selection for retention of photosynthetic genes in the parasitic plant genus *Cuscuta*

**DOI:** 10.1186/1471-2229-7-57

**Published:** 2007-10-24

**Authors:** Joel R McNeal, Jennifer V Kuehl, Jeffrey L Boore, Claude W de Pamphilis

**Affiliations:** 1 Department of Plant Biology, University of Georgia, Athens, GA 30602, USA; 2 Department of Biology, Huck Institutes of the Life Sciences, and Institute of Molecular Evolutionary Genetics, The Pennsylvania State University, University Park, Pennsylvania 16802-5301, USA; 3 DOE Joint Genome Institute and Lawrence Berkeley National Laboratory, Walnut Creek, California 94598, USA; 4 Genome Project Solutions, Hercules, CA 94547, USA

## Abstract

**Background:**

Plastid genome content and protein sequence are highly conserved across land plants and their closest algal relatives. Parasitic plants, which obtain some or all of their nutrition through an attachment to a host plant, are often a striking exception. Heterotrophy can lead to relaxed constraint on some plastid genes or even total gene loss. We sequenced plastid genomes of two species in the parasitic genus *Cuscuta *along with a non-parasitic relative, *Ipomoea purpurea*, to investigate changes in the plastid genome that may result from transition to the parasitic lifestyle.

**Results:**

Aside from loss of all *ndh *genes, *Cuscuta exaltata *retains photosynthetic and photorespiratory genes that evolve under strong selective constraint. *Cuscuta obtusiflora *has incurred substantially more change to its plastid genome, including loss of all genes for the plastid-encoded RNA polymerase. Despite extensive change in gene content and greatly increased rate of overall nucleotide substitution, *C. obtusiflora *also retains all photosynthetic and photorespiratory genes with only one minor exception.

**Conclusion:**

Although *Epifagus virginiana*, the only other parasitic plant with its plastid genome sequenced to date, has lost a largely overlapping set of transfer-RNA and ribosomal genes as *Cuscuta*, it has lost all genes related to photosynthesis and maintains a set of genes which are among the most divergent in *Cuscuta*. Analyses demonstrate photosynthetic genes are under the highest constraint of any genes within the plastid genomes of *Cuscuta*, indicating a function involving RuBisCo and electron transport through photosystems is still the primary reason for retention of the plastid genome in these species.

## Background

Parasitic plants offer excellent opportunities to study changes in genome evolution that accompany the switch from an autotrophic to a heterotrophic lifestyle, a transition that has occurred many times over the course of evolution. Within angiosperms, the ability to obtain nutrition through direct attachment to a host plant has evolved at least a dozen times [1] with many additional instances of plants obtaining most or all of their nutrition through specific mycotrophic fungal interactions [2,3]. While approximately 90% of genes involved in photosynthesis have been transferred to the nuclear genome over the course of chloroplast evolution since divergence from free-living cyanobacterial relatives [4], these nuclear genes are often difficult to study in non-model organisms. Widespread gene and genome duplication often makes inference of orthology among nuclear genes difficult, and rate acceleration in ribosomal loci of some parasitic plants suggests that the sequences of nuclear genes may be too divergent to amplify through standard PCR [5]. By contrast, genes remaining on the plastid chromosome evolve more slowly than nuclear genes and exist as single, readily identifiable orthologs in each plastome, although the plastid chromosome itself is in high copy number per cell [6].

Many species of parasitic plants retain the ability to photosynthesize, and aside from a supplemental connection to the roots of a host, otherwise resemble fully autotrophic plants in habit [7]. Others, however, display increased dependency on their hosts, often to the extent of becoming fully heterotrophic and nonphotosynthetic. Such plants are often deemed "holoparasites", and one such species, *Epifagus virginiana *(Beechdrops, Orobanchaceae) is the only parasitic plant whose full plastid genome has been sequenced to date [8]. Its plastid genome is reduced to less than half the size of that in normal angiosperms due to ubiquitous gene loss, including all photosynthetic and photorespiratory genes, some ribosomal protein genes, many tRNA genes, and genes for plastid-encoded polymerase [8,9]. Despite such drastic changes, plastid transcription and intron splicing still occur [9,10], presumably for the purpose of producing the four remaining proteins not related to transcription or translation. Smaller scale studies show similar or less genome reduction in related species [11-14]. For some holoparasitic lineages, existence of a functional plastid genome remains to be proven, although preliminary evidence suggests extremely divergent plastid genomes may occur in the families Balanophoraceae, Cytinaceae, Hydonoraceae, and Cynomoriaceae [15,16].

A large number of studies on plastid function have been performed involving members of the parasitic genus *Cuscuta*, derived from within the otherwise autotrophic Morning Glory Family (Convolvulaceae, order Solanales, class Asteridae). Plastid ultrastructure and gene content are quite variable between different taxa [17], and over 150 species exist in this widespread and recognizable genus [18]. Unlike *Epifagus *and other root-parasitic Orobanchaceae, *Cuscuta *is a twining vine with no roots at maturity. Instead, it sends its shoot like feeding organs, haustoria, directly into the stems of its hosts to invade the vasculature and obtain all necessary water and other nutrients. Leaves are reduced to vestigial scales. Despite an obligate reliance upon their hosts, many *Cuscuta *species show some green color, at least in their inflorescences and, particularly, in maturing ovules. Machado and Zetsche demonstrated the presence of RuBisCo, chlorophyll, and low levels of carbon fixation in *Cuscuta reflexa*, a member of subgenus *Monogyna *[19]. Additionally, although all NADH dehydrogenase (*ndh*) genes were either undetectable or nonfunctional [20], other genes related to photosynthesis appeared to be present in functional form [21]. In this species, green plastids of normal function are localized to a ring of cells between the stem pith and cortex that are isolated from atmospheric gas exchange, indicating photosynthesis may occur in this species using recycled respiratory CO_2 _[22] despite an altered xanthophyll cycle in its light-harvesting complex [23].

Dot blots using poly-A selected RNA from *C. reflexa *as a probe also showed positive hybridization to some of the 101 tobacco genes in tobacco, although whether these results actually represent nuclear transcribed copies of the plastid genes, polyadenylated plastid transcripts, or leakage of non-polyadenylated plastid transcripts through cDNA production steps is unclear [24]. A different situation exists in *Cuscuta pentagona *(subgenus *Grammica*), which lacks the ring of photosynthetic cells observed in the stems of *C. reflexa*, but possesses what appear to be photosynthetically capable plastids with immunodetectable RuBisCo, photosystem, and light-harvesting proteins in proper locations within the plastids in green tissues of seedlings and adult plants [25]. Other species within subgenus *Grammica *show a range of *rbcL *transcript levels, from low to none [17], and sampled members of this subgenus lack promoters for plastid-encoded polymerase upstream of the *rrn16 *and *rbcL *genes, although transcription of *rbcL *still occurs from nuclear-encoded polymerase promoter sites in both cases [26]. Conflicting evidence exists for *Cuscuta europaea *(subgenus *Cuscuta*), which has been described as lacking chlorophyll and detectable *rbcL *protein [19], yet still possesses green color and more typical plastid sequences, including *rbcL*, than members of subgenus *Grammica *[27]. Other minor changes have been detected in the plastid genome of *Cuscuta sandwichiana*, such as deletion of introns within *ycf3*, constriction of the inverted repeat to exclude *rpl2*, *rpl23*, and *trnI*, loss of *trnV*-UAC, and reduction in size of *ycf2*; slight changes to the end of *atpB*, size reduction of the *trnL *intron, and deletion of the *rpl2 *intron are shared with other, non-parasitic Convolvulaceae, and occurred before the evolution of parasitism in *Cuscuta *[28].

In this study, we test if significant changes to the plastid genome have occurred prior to the evolution of parasitism, if previously published observations of plastid genome evolution in *Cuscuta *apply to other members of the genus, if differences in chlorophyll content and distribution between *Cuscuta *species parallel differences in plastid genome content, whether plastid genes retained in *Cuscuta *are still evolving under strong purifying selection, and whether plastid gene retention and selective constraint suggest a photosynthetic function for plastids in this parasitic genus. To do so, we sequenced the full plastid genomes of two species of *Cuscuta *and a close photosynthetic relative, *Ipomoea purpurea *(Common Morning Glory). *Ipomoea *is a member of the Convolvuloideae clade, which has been shown as the most likely sister group to *Cuscuta *in a number of studies [27,29-31]. *Cuscuta exaltata*, a member of subgenus *Monogyna *with visible chlorophyll distributed throughout the stems and inflorescences, and *Cuscuta obtusiflora*, a member of subgenus *Grammica *that usually only exhibits green pigmentation in inflorescences, fruits, starved seedlings and stressed stem tips, were chosen to represent *Cuscuta*. We examined overall rates of substitution and changes in selective constraint by comparing rates of synonymous and nonsynonymous substitution for all plastid genes and across functionally defined classes of genes to determine if photosynthetic genes remain the most highly conserved in the plastid genome and whether relaxation of functional constraint precedes gene losses both before and after the evolution of parasitism in this lineage. We also tested whether patterns of transfer RNA loss, changes in intergenic regions, and rates of substitution parallel those seen in the completely nonphotosynthetic *Epifagus virginiana*. Finally, we use the cumulative evidence of photosynthetic localization, specific gene loss, and strong functional constraint of specific genes to suggest a photosynthetic function of the plastid genome unrelated to the Calvin Cycle in *Cuscuta *and perhaps other parasitic plants as well.

## Results and Discussion

### Plastid Genome Size and Inverted Repeat Structure

**Figure 1:**
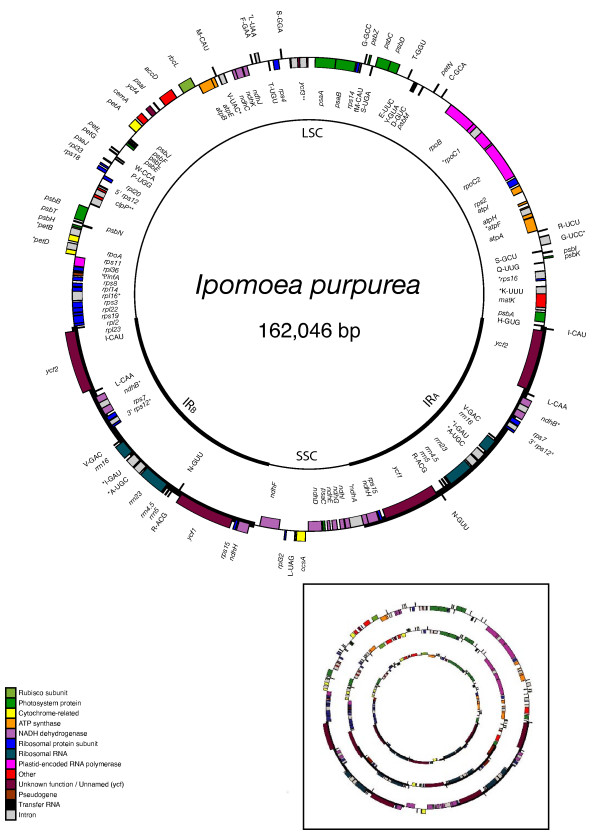
**Circular map of the complete plastid genome of *Ipomoea purpurea***. The genome comprises an 88,172 bp LSC, a 12,110 bp SSC, and two 30,882 bp IRs. Position one of the annotated sequence begins at the LSC/IR_A _junction and increases numerically counterclockwise around the genome. Genes on the inside of the circle are transcribed clockwise, those on the outside, counterclockwise. Asterisks mark genes with introns (2 asterisks mark genes with 2 introns), Ψ indicates a pseudogene. INSET-Genomes scaled to relative size: *Ipomoea *(outermost), *Cuscuta exaltata *(middle), and *C. obtusiflora *(innermost).

**Figure 2:**
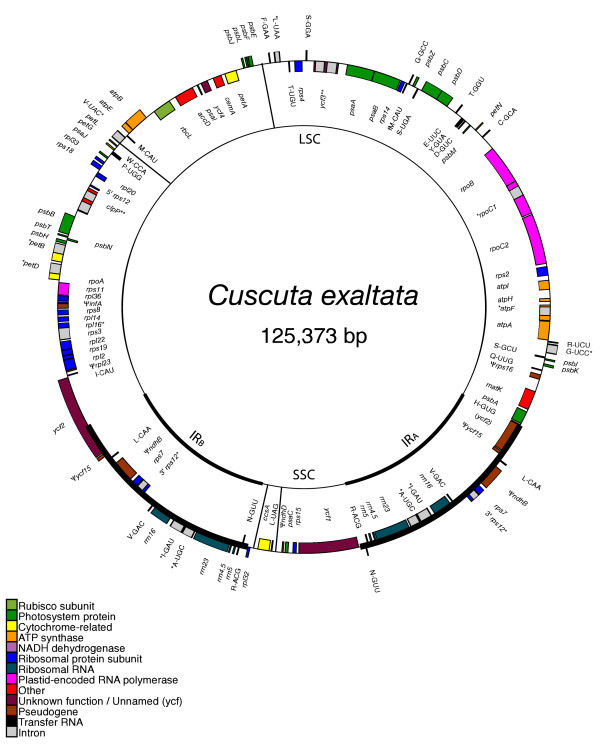
**Circular map of the complete plastid genome of *Cuscuta exaltata***. The genome comprises an 82,721 bp LSC and a 9,250 bp SSC separated by two 16,701 bp IRs. Inversion end-points are shown with lines connecting the inner circle to the outer. Position one of the annotated sequence begins at the LSC/IR_A _junction and increases numerically counterclockwise around the genome. Genes are denoted as in Figure 1.

**Figure 3:**
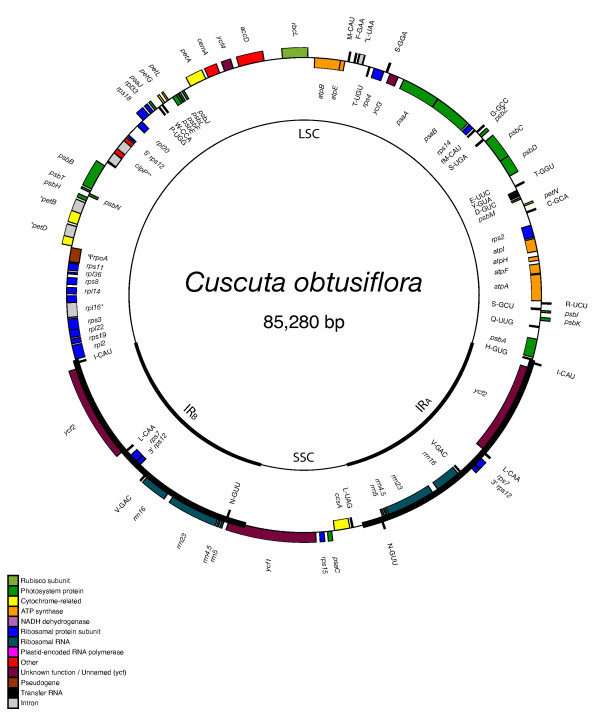
**Circular map of the complete plastid genome of *Cuscuta obtusiflora***. The genome comprises a 50,201 bp LSC and a 6,817 bp SSC separated by 14,131 bp IRs. Position one of the annotated sequence begins at the LSC/IR_A _junction and increases numerically counterclockwise around the genome. Genes are denoted as in Figure 1.

The three plastid genomes presented here all have a pair of large, inverted, identical repeat sequence (IR) separated from each other by a large single copy and small single copy region (LSC and SSC) on either end, as is the case for practically all plant plastid genomes [32]. However, considerable length variation exists between these three plastid genome sequences, with the smallest genome, *Cuscuta obtusiflora*, barely half the size of that in *Ipomoea purpurea *(85,280 base pairs versus 162,046 bp). *Cuscuta exaltata *is intermediate in size at 125,373 bp (See figs. 1, 2, 3). The plastid genome of *Ipomoea *is slightly larger than that of *Nicotiana tabacum *(155,939 bp), largely through expansion of the IR region into the SSC region (fig. 1). While the IR of *Nicotiana *barely extends into *ycf1*, the IR of *Ipomoea *includes the entire *ycf1 *gene, *rps15*, *ndhH*, and a short fragment of the first exon of *ndhA*. By contrast, the LSC end of the IR is slightly constricted, not including *rpl2 *and *rpl23 *as it does in *Nicotiana*. Previous estimates of plastid genome sizes in Convolvulaceae based upon relative size of restriction fragments using Southern blots with tobacco plastid fragments as a probe showed a similar, stepwise trend in plastid genome size reduction from a non-parasite to a member of *Cuscuta *subgenus *Monogyna *to various other species [24]. In that study, a non-parasitic member Convolvulaceae very closely related to *Ipomoea*, *Convolvulus arvensis*, gave a plastid genome size estimate 24 kbp larger than our *Ipomoea purpurea *sequence (186 kb vs. 162 kb). The rather large discrepancy could be due to an even larger increase in the IR size in *Convolvulus *relative to tobacco than is seen in *Ipomoea*, or it could simply reflect an inability to properly detect IR boundaries using the rough restriction fragment analysis employed in that study. *Cuscuta reflexa *gave a plastid genome size estimate of 122 kbp in that study, which matches up well with the 125 kb size we sequenced for *Cuscuta exaltata*, also in subgenus *Monogyna*. Estimates of other *Cuscuta *species ranged from 81 kbp to 104 kbp, although apparent misidentification of some of the species in that study makes further phylogenetic comparison difficult [33].

### Plastid Gene Content

**Figure 4:**
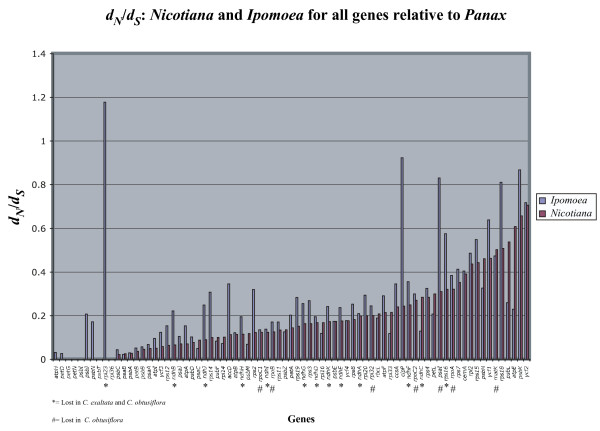
**Pairwise *d*_*N*_/*d*_*S *_of *Nicotiana *and *Ipomoea *vs. *Panax ginseng *for all shared protein-coding genes**. Genes are ranked left to right by increasing *d*_*N*_/*d*_*S *_for *Nicotiana*. Genes lost in *Cuscuta exaltata *and *C. obtusiflora *are indicated below the graph.

Gene content in *Ipomoea *is decidedly similar to that in *Nicotiana *and *Atropa*. These three taxa, along with both *Cuscuta *species, lack an intact *infA *[34], indicating this gene loss probably occurred prior to the divergence of Solanaceae from Convolvulaceae, both in the order Solanales. This is not surprising, as *infA *has been lost from the plastid many times in angiosperm evolution [35]. A second gene, *ycf15*, is lost across Convolvulaceae taxa sequenced in this study but is present in Solanaceae and outgroups [34,36,37]. However, the function of this gene is not known, and the effect of its loss in Convolvulaceae is difficult to interpret. A third gene, *rpl23*, is clearly a pseudogene in *Cusucta exaltata *and is lost completely in *C. obtusiflora*, but it is not clear whether it is functional in *Ipomoea*. Although a full length open reading frame exists in *Ipomoea *for *rpl23*, it contains two frameshift mutations and an extension of the 3' end. The gene also does not appear to be evolving under purifying selective constraint as in *Nicotiana *(see fig. 4), further indicating it may be a pseudogene, although tests of expression will be necessary to confirm this. Despite being a component of the plastid translational apparatus, the expendability of this ribosomal protein gene subunit in its plastid location is supported by its loss from the plastid genome *Spinacia *as well [37]. A gene found thus far only in members of Solanaceae, *sprA *[34], is not found in any of the sequenced Convolvulaceae genomes, indicating presence of this gene in the plastome is restricted to Solanaceae.

One gene that was surprisingly found in all three Convolvulaceae plastid genomes is *ycf1*, a large gene of unknown function previously reported as missing in *Cuscuta *and three other Convolvulaceae [38]. That study used Southern Blot hybridizations to screen for gene presence; *ycf1 *is still present as the second largest open reading frame in the plastid genome, but is extremely variable in size between the two *Cuscuta *species and is greatly elongated in *Ipomoea*, possesses numerous large indels, and is difficult to align with other species at the protein level in some regions These factors likely explain the negative hybridizations previously observed. Although it is one of the least conserved genes in both *Cuscutas *and *Ipomoea*, it is still apparently evolving under selective constraint as a functional gene. As is the case for *ycf15*, interpreting consequences of the extreme divergence of this gene in Convolvulaceae awaits full knowledge of its function.

Gene loss is much more prominent in the two *Cuscuta *species than *Ipomoea*. All genes lost in *C. exaltata *are also lost in *C. obtusiflora*, and are most parsimoniously assumed to be lost in the common ancestor of both species. Most notable of these losses are the *ndh *genes, all of which are fully lost from the plastid or are pseudogenes in *Cuscuta*. This confirms the PCR and blot data collected for *Cuscuta reflexa *that suggested all *ndh *genes were missing, highly altered, or translocated in that species [21] as well as negative PCR and sequence results from other species [28]. All *ndh *genes are also lost from the plastid in *Epifagus *[9], indicatingevolution of parasitism may facilitate loss of these genes or movement to the nuclear genome. Although *ndh *genes are retained in most photosynthetic plants, they are also lost from the chloroplast genome of *Pinus *[39], indicating their presence in the plastid genome is not necessary for photosynthesis even in fully autotrophic plants. Both *Cuscuta *species also lack a functional *rps16 *gene in the plastid, although *C. exaltata *contains a pseudogene with portions of both exons and the group II intron present between them. A final gene loss from both *Cuscuta *plastomes that is also reported in *C. reflexa *is the loss of *trnK*-UUU [40]. As is the case for *Epifagus*, *C. exaltata *retains the open reading frame, *matK*, contained within the intron of that tRNA. A deletion within the *trnV *-CAU intron also reported in *C. reflexa *[21], and similar to that seen in *Orobanche minor*, may hypothetically disrupt its splicing [13], but because both exons remain intact in these species, we hesitate to call it a pseudogene in *C*. *exaltata *without experimental evidence. Aside from these gene losses, plastid genome content of *C. exaltata *is identical to that in *Ipomoea *and includes a full set of genes presumably necessary for photosynthesis.

### Plastid Genome Rearrangements

Structurally, the plastid genome of *C. exaltata *has undergone a number of changes relative to *Ipomoea *and *Nicotiana*. The LSC end of the IR is constricted in both *Cuscuta *species, but it has apparently re-extended to include a few nucleotides of *trnH*-GUG (4 nucleotides in *C. exaltata*, 6 in *C. obtusiflora*). As in *Ipomoea*, the first full gene in the LSC end of the IR in *C. obtusiflora *is *trnI*-CAU. However, the IR constriction is much more dramatic in *C. exaltata*, with *rpl2*, *trnI*, and over half of *ycf2 *falling outside the IR (fig. 2). Putative loss of these genes in *C*. *reflexa *detected by PCR [40] is likely an artifact of this constriction rather than a deletion, as the primers used in that study would have shown similar results for *C. exaltata *and not amplified the opposite LSC/IR junction at which these genes actually do exist. The IR has not extended substantially into the SSC in *Cuscuta *as in *Ipomoea*. In fact, *C. exaltata *is somewhat contracted relative to *Nicotiana *and ends slightly before the start codon of *ycf1*. Like *Nicotiana*, the IR of *C. obtusiflora *contains a portion of the 5' end of *ycf1*. Two segmental inversion events are observed in *C. exaltata*. One inversion occurs from *trnV*-UAC to *psbE *in the LSC region, the other in the SSC encompassing only two genes, *ccsA *and *trnL*-UAG. Both of these inversions border on regions that once contained *ndh *genes. Extensive noncoding pseudogene sequence may have helped ameliorate accumulation of repeat sequences that could promote inversion. Perhaps not coincidentally, the only inversion observed in *Epifagus *is *trnL*-UAG in the SSC [8].

### Plastid Genome Changes in *C. obtusiflora*

The plastid genome of *Cuscuta obtusiflora *surprisingly lacks any structural rearrangements relative to *Nicotiana *and *Ipomoea*. Unlike *C. exaltata*, *C. obtusiflora *lacks extensive pseudogene sequence and may have purged such unused DNA from its plastome before sequence motifs conducive to inversion events had time to develop. Gene loss, on the other hand, is much more rampant within *C. obtusiflora *(Table 1). In addition to the genes previously discussed for *C. exaltata*, *C. obtusiflora *has lost a third ribosomal protein gene, *rpl32*, and five additional tRNAs. Also lost are all subunits of the plastid-encoded RNA polymerase (*rpo*), and the intron maturase *matK*, the loss of which parallels loss of all group IIA introns from the genome as well, as previously reported [41]. Blot data and negative PCR results have suggested loss of plastid *rpo *genes from other species within subgenus *Grammica *as well [26,28], although the *rrn *gene cluster and *rbcL *gene appear to still be transcribed from nuclear-encoded polymerase in at least some species [42]. Despite such extensive gene loss from the plastome, *C. obtusiflora *retains all plastid genes directly involved in photosynthesis within the chloroplast, including all *atp *genes, all *pet *genes, *rbcL*, and all *psa *and *psb *genes, with the exception of *psaI*. This gene is one of the smallest in the plastome (36 codons or less), although it is highly conserved across land plants. Losses of *trnV *and two introns within *ycf3 *reported for another member of subgenus *Grammica*, *Cuscuta sandwichiana *[28], are also present in *Cuscuta obtusiflora*.

**Table 1:** Plastid gene loss relative to *Panax ginseng*

Gene Type	*Ipomoea purpurea*	*Cuscuta exaltata*	*Cuscuta obtusiflora*
NADH dehydrogenase		*ndhA*, Ψ *ndhB*, *ndhC*, Ψ *ndhD*, *ndhE*, *ndhF*, *ndhG*, *ndhH*, *ndhI*, *ndhJ*, *ndhK*	*ndhA*, *ndhB*, *ndhC*, *ndhD*, *ndhE*, *ndhF*, *ndhG*, *ndhH*, *ndhI*, *ndhJ*, *ndhK*,
Photosystem Protein			*psaI*
Ribosomal Protein	(Ψ *rpl23*?)	Ψ *rpl23*, Ψ *rps16*	*rpl23*, *rpl32*, *rps16*
Transfer-RNA		*trnK*-UUU	*trnA*-UGC,*trnG*-UCC, *trnI*-GAU,*trnK*-UUU, *trnR*-ACG†,*trnV*-UAC
RNA polymerase			Ψ *rpoA, rpoB, rpoC1*, *rpoC2*
Initiation factor	Ψ infA*†	Ψ infA*†	infA*†
Unknown	*ycf15*	Ψ *ycf15*	*ycf15*
Intron maturase			*matK*†

* Also nonfunctional in *Nicotiana tabacum *and *Atropa belladonna*

† Still present in *Epifagus virginiana*

### Selective Constraint in Plastid Genes

With these three new full plastid genome sequences, we tested whether substantial changes in selective pressure of genes, particularly those lost in *Cuscuta*, occurred prior to evolution of parasitism in this lineage. After calculating corrected distances of nonsynonymous nucleotide substitution per nonsynonymous site (*d*_*N*_) and synonymous substitution per synonymous site (*d*_*S*_) for all genes in *Ipomoea *and *Nicotiana *relative to a common outgroup, *Panax*, an interesting trend toward relaxed selection in the genome of the fully autotrophic *Ipomoea *was revealed (fig. 4). Of 77 protein-coding genes shared between the two taxa, 56 (72.7%) have a higher *d*_*N*_/*d*_*S *_in *Ipomoea *than in *Nicotiana*, with only 15 genes showing higher *d*_*N*_/*d*_*S *_in *Nicotiana *(6 genes were indistinguishable or had *d*_*N*_/*d*_*S *_< 0.01 in both taxa). Furthermore, 12/13 genes lost in both *Cuscuta *species had higher *d*_*N*_/*d*_*S *_in *Ipomoea*, indicating these genes may have already been under relaxed selection prior to the evolution of parasitism in Convolvulaceae. Using likelihood methods, all previously defined classes of genes (*atp*, *pet*, *ps*, *rp*, *rpo*, and *ndh*) with the exception of *pet *showed significantly greater overall rates of substitution in *Ipomoea *than in *Nicotiana *in pairwise relative rates test using *Panax *as an outgroup (Table 2). Analysis of the combined set of *ndh *genes revealed that the ratio of nonsynonymous substitution rates to synonymous substitution rates (R) on the branch leading to *Ipomoea *is much higher than in the previous branch in the tree leading to Solanales leading to an extremely significant difference in the likelihood of the tree when left unconstrained (p < 0.0001, Table 3), suggesting relaxed selection in *ndh *genes probably began before the advent of parasitism.

**Table 2:** Results of pairwise relative rates and relative ratio tests

A			
Pairwise Relative Rates Tests			
Taxa compared	*C. exaltata *vs. *C. obtusiflora*	*C. exaltata *vs. *Ipomoea*	*Ipomoea *vs. *Nicotiana*
Outgroup	*Ipomoea*	*Nicotiana*	*Panax*
atp	***	***	0.00377*
pet	***	***	0.257
ps	***	***	***
rp	***	***	***
rpo		***	***
ndh			***
B			
Relative Ratio Tests			
Gene Classes Compared	*atp *vs. *pet*	*atp *vs. *ps*	*atp *vs. *rp*
*dN*	0.246	0.00634*	0.0231
*dS*	0.186	0.0254	0.116543
Gene Classes Compared	*pet *vs. *ps*	*pet *vs. *rp*	*ps *vs. *rp*
*dN*	0.0783	0.0554	***
*dS*	0.983	0.911	0.27

*p < 0.01, **p < 0.001, ***p < 0.0001. All significant results remain significant after sequential Bonferroni tests as described in Rice, W.R. 1989. Evolution. 43: 223–225.

**Figure 5:**
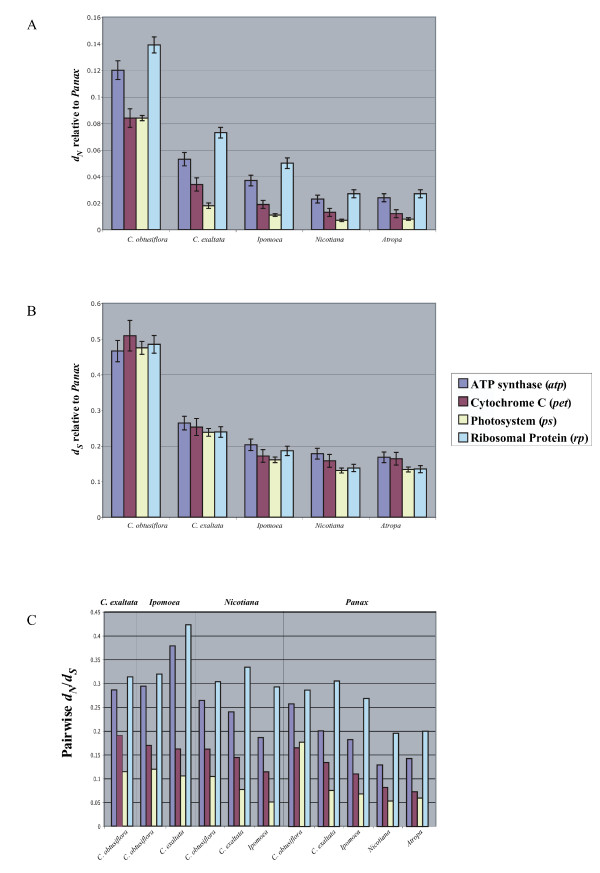
Rates of substitution and selection across 4 functionally-defined classes of genes. A- *d*_*N *_estimates and standard errors vs *Panax *for *Atropa*, *Nicotiana*, *Ipomoea*, *C. exaltata*, and *C. obtusiflora*. B-*d*_*S *_vs *Panax *for the same taxa. C. Pairwise *d*_*N*_/*d*_*S *_for the same taxa vs. *Panax*; *Ipomoea*, *C. exaltata*, and *C. obtusiflora *vs. *Nicotiana*; *C. exaltata *and *C. obtusiflora *vs. *Ipomoea*, and *C. exaltata *vs. *C. obtusiflora*.

Pairwise relative rates tests also show significant overall rate differences between *Ipomoea *and *Cuscuta exaltata *as well as between the two *Cuscuta *species for all types of genes (Table 2). We next wanted to test whether ratios of overall selection between classes of genes remaining in *Cuscuta *are similar to autotrophic taxa. Figure 5 shows how patterns of synonymous and nonsynonymous substitution vary between sampled Solanalean taxa relative to *Panax *for the various classes of genes in the plastome. While there are minor changes in synonymous rates between different gene classes, relative ratio tests of synonymous rates for the tree topologies of each gene class yielded no significant differences (Table 2). However, nonsynonymous rate values for *ps *genes were significantly different from both *atp *and *rp *genes, and there were lower nonsynonymous rates and R for *pet *and *ps *genes in all pairwise comparisons performed (fig. 5b and 5c). The trend in *Cuscuta *is clearly symmetrical to other taxa; all classes of genes appear to be evolving under strong negative selection with R much lower than 1, and photosystem and *pet *genes remain the most highly conserved, even in the rapidly evolving *C. obtusiflora *genome. Despite the loss of *psaI *in *C. obtusiflora*, selective constraint on the plastid genome of both *Cuscuta *species strongly suggests that a photosynthetic process remains the primary purpose of their plastid genomes.

**Figure 6:**
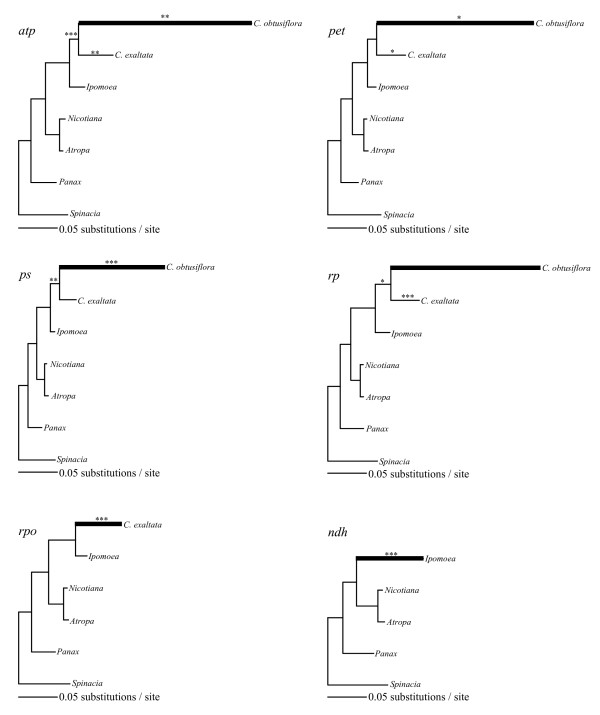
**Phylogenetic trees created using Maximum Likelihood GTR+gamma for each functionally defined gene class**. Branches with significantly higher (LRT, p < 0.01) rates of synonymous substitution per site are thickened. Branches with significantly higher *d*_*N*_/*d*_*S *_are marked with one (p < 0.01), two, (p < 0.001), or three asterisks (p < 0.0001). Values of *d*_*S *_and *d*_*N*_/*d*_*S *_on relevant branches are given in Table 3.

Although plastid genes in *Cuscuta *are still evolving under strong negative selection, the data show that they are somewhat relaxed compared to their fully autotrophic relatives. Figure 6 shows phylograms for each of the previously discussed gene classes with significant increases in synonymous rates and R as determined by LRTs indicated on the branches. The overall synonymous rate for *C. obtusiflora *varies between 5 and 8 times that of the branch leading to Convolvulaceae across the four classes of genes for which it could be studied, while that of *C*. *exaltata *is nearly identical (Table 3). These highly accelerated substitution rates in *C. obtusiflora *could be the result of shorter generation time, damage to repair machinery allowed by relaxed selective constraint, or, alternatively, could result from a lower organismal or plastid genome population size [43]. Strongly negative selective pressure in *C. obtusiflora*, particularly in *ps *and *pet *genes, occurring in spite of highly accelerated rates of nucleotide substitution further supports the idea that *C. obtusiflora *must be utilizing its photosynthetic genes for some purpose important to the plant. This is particularly fascinating considering full loss of plastid-encoded polymerase. While *Epifagus virginiana *has been shown to perform transcription of ribosomal and various other protein coding genes in the absence of plastid *rpo *genes [9,10], this phenomenon is unknown from any photosynthetic plant. In large part, plastid polymerase performs the transcriptional duties for photosynthetic genes in typical green plants, but a dramatic shift seems to have occurred in *Cuscuta *toward imported nuclear polymerase transcription of all genes. Many plastid genes are known to be transcribed by both polymerases [44], and whether or not autotrophic relatives of *Cuscuta obtusiflora *already possess the ability to transcribe all genes with imported nuclear polymerase or whether novel promoters and transcription factor binding sites evolved rather recently remains to be seen.

**Table 3:** Rates of synonymous substitution and ratio of nonsynonymous to synonymous substitution

*atp*	*R*_*N*_*/R*_*S*_	*R*_*S*_	Synonymous rate increase (× Convolvulaceae)
Globally constrained	0.184	0.205	
Solanales	0.0885	0.136	
Convolvulaceae	0.133	0.189	
*Cuscuta*	0.821***	0.0206	
*C. exaltata*	0.264**	0.216	1.14
*C. obtusiflora*	0.231**	0.959***	5.07
*pet*			
Globally constrained	0.114	0.208	
Solanales	0.0381	0.131	
Convolvulaceae	0.0907	0.135	
*Cuscuta*	0.0606	0.127	
*C. exaltata*	0.245*	0.16	1.19
*C. obtusiflora*	0.154*	1.037***	7.68
*ps*			
Globally constrained	0.0728	0.191	
Solanales	0.0402	0.106	
Convolvulaceae	0.046	0.159	
*Cuscuta*	0.138**	0.0847	
*C. exaltata*	0.0859	0.17	1.07
*C. obtusiflora*	0.103***	0.91***	5.72
*rp*			
Globally constrained	0.227	0.184	
Solanales	0.0988	0.106	
Convolvulaceae	0.267	0.157	
*Cuscuta*	0.314*	0.0959	
*C. exaltata*	0.390***	0.148	0.94
*C. obtusiflora*	0.232	0.901***	5.74
*rpo*			
Globally constrained	0.231	0.175	
Solanales	0.144	0.163	
Convolvulaceae	0.218	0.211	
*C. exaltata*	0.435***	0.253***	1.2
*ndh*			
Globally constrained	0.19	0.223	
Solanales	0.181	0.114	
*Ipomoea*	0.284***	0.401***	

Significance of rate differences determined by comparing fully constrained tree likelihood to tree with specified node unconstrained. *p < 0.01, **p < 0.001, ***p < 0.0001. All significant results remain significant after sequential Bonferroni tests as described in Rice, W.R. 1989. Evolution.43: 223–225.

### Plastid Genome Differences in *Cuscuta *versus *Epifagus virginiana*

Although *Epifagus virginiana *has undergone a similar downsizing of its plastid genome, it and *Cuscuta *are quite different in a number of ways, most obviously in that *Cuscuta *retains a seemingly functional set of photosynthetic genes while *Epifagus *has lost all such genes. With the loss of *rpo *genes in both taxa, we investigated whether both taxa show similar patterns of deletion in intergenic regions, which should contain plastid promoters, transcription-factor binding sites, and other motifs no longer necessary in a nuclear-transcribed plastome. Overall, *Epifagus *has 22 fewer protein coding genes and 7 fewer tRNA genes than *C. obtusiflora*. While the plastid genome size of *Epifagus *(70,028 bp) is over 15 kilobases smaller than that of *C*. *obtusiflora*, this is actually less than would be expected given such a dramatic difference in overall gene content. In 63 non-coding, intergenic regions between homologous functional genes in both *Cuscuta *species, *Ipomoea*, and *Nicotiana*, *C. obtusiflora *(11714) has undergone a 49% overall decrease in length relative to *Nicotiana *(22,996 bp), perhaps largely due to a deletion of plastid polymerase and transcription factor binding sites. *C. exaltata *has decreased 16% over the same area, and *Ipomoea *only 1%. Over the 16 intergenic regions shared by *Epifagus*, *C. obtusiflora *has decreased by 33% relative to *Nicotiana*, while *Epifagus *has only decreased by slightly over 3% (values in Table 4A). Likewise, in 3 regions for which conserved functional genes flanking regions containing homologously defunct genes could be compared between *Epifagus *and *C*. *obtusiflora*, *Epifagus *exhibits a 32% total decrease in size relative to the full length sequences containing functional genes in *Nicotiana*, while *C. obtusiflora *is 85% shorter (Table 4B). The IR of *Epifagus *is almost the same length as that of a normal angiosperm, while its SSC and LSC regions are the sites of practically all of its gene loss. *Cuscuta obtusiflora *has extensive deletion in those areas too, but also exhibits a significant contraction of the IR, largely through pseudogene loss relative to *Epifagus*. While *Cuscuta obtusiflora *almost completely lacks pseudogene sequences, *Epifagus *retains a fair number of them. Coupled with various intron losses, the plastid genome of *C. obtusiflora *is much more streamlined than that of *Epifagus*.

**Table 4:** (A) Intergenic distance between shared, intact coding sequence and (B) Shared pseudogene sequence relative to Nicotiana

A										
Region (flanking genes)		*Epifagus*		*C. obtusiflra*		*C. exaltata*		*Ipomoea*		*Nicotiana*
*rpl20-rps12*		745		720		799		814		811
*trnN-ycf1*		323		113		537		402		328
*rrn4.5-rrn5*		233		83		218		241		256
*trnW-trnP*		225		153		74		169		164
*rps18-rpl20*		213		102		244		257		205
*rrn23-rrn4.5*		188		80		100		101		101
*trnS-rps4*		182		119		310		313		333
*rpl16-rps3*		162		79		159		166		146
*trnD-trnY*		151		54		106		106		108
*rps12-clpP*		138		81		187		187		141
*trnfM-rps14*		133		126		152		144		152
*rpl33-rps18*		115		124		185		193		189
*rps11-rpl36*		108		103		100		94		104
*trnY-trnE*		80		68		70		55		59
*rps19-rpl2*		62		96		64		51		63
*rps7-rps12*		47		48		53		53		53
*trnQ-psbK*				350		360		370		347
*trnR-atpA*				101		87		108		126
*atpA-atpF*				66		61		59		54
*atpF-atpH*				202		294		353		401
*atpH-atpI*				289		744		1113		1160
*atpI-rps2*				79		412		239		229
*trnC-petN*				347		709		928		670
*petN-psbM*				353		497		1197		1138
*psbM-trnD*				94		459		660		1072
*trnE-trnT*				200		585		747		848
*trnT-psbD*				636		868		1322		1218
*psbC-trnS*				107		283		250		242
*trnS-psbZ*				133		262		351		362
*psbZ-trnG*				168		285		278		278
*trnG-trnfM*				89		100		270		227
*rps14-psaB*				98		100		128		125
*psaB-psaA*				19		28		28		28
*psaA-ycf3*				349		724		790		752
*ycf3-trnS*				166		576		829		1716
*rps4-trnT*				240		425		457		371
*trnT-trnL*				370		660		875		710
*trnL-trnF*				113		357		371		356
*trnM-atpE*				172		209		211		224
*atpB-rbcL*				401		799		414		817
*rbcL-accD*				1053		584		899		767
*ycf4-cemA*				296		517		616		222
*cemA-petA*				170		318		220		230
*petA-psbJ*				458		642		909		1071
*psbJ-psbL*				115		167		137		127
*psbL-psbF*				40		25		25		25
*petL-petG*				171		257		230		184
*petG-trnW*				50		124		131		134
*trnP-psaJ*				125		392		543		438
*psaJ-rpl33*				114		356		460		434
*clpP-psbB*				253		565		511		445
*psbB-psbT*				110		163		203		203
*psbT-psbN*				60		62		71		82
*psbN-psbH*				122		110		116		111
*psbH-petB*				120		130		133		129
*petB-petD*				132		195		209		190
*rpl36-rps8*				206		542		490		439
*rps8-rpl14*				178		170		278		171
*rpl14-rpl16*				107		145		105		124
*rpl22-rps19*				90		62		68		56
*trnV-rrn16*				116		220		221		227
*trnL-ccsA*				119		158		124		103
*rps15-ycf1*				218		207		369		400
# bp				11714		19353		22762		22996
% change				-49.06%		-15.84%		-1.02%		
Only those shared w/*E. v*.		3105		2149		3358		3346		3213
% change		-3.36%		-33.12%		4.51%		4.14%		
B										
		*Epifagus*	% change		*C. obtusiflra*	% change		*Nicotiana*		
*rrn16-rrn23*		2009	-3.32%		341	-83.59%		2078		
*rps7-trnL*		1322	-56.37%		307	-89.87%		3030		
*trnI-rpl2*		471	1.29%		168	-63.87%		465		
		3802	-31.78%		816	-85.36%		5573		

Pseudogene sequence is defined as intergenic spacer regions between conserved genes that once contained one or more genes no longer present in functional form in either species.

**Figure 7:**
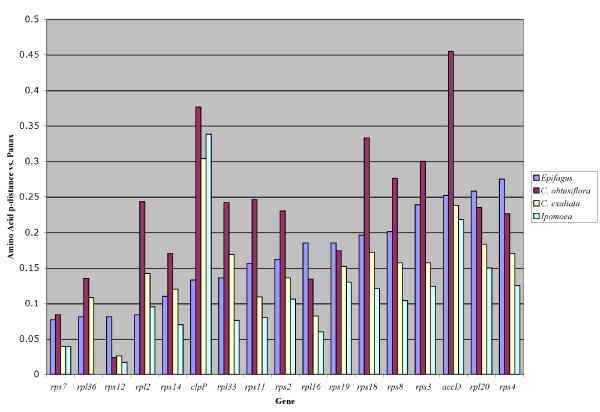
**Amino acid *p*-distance for *Epifagus*, *Ipomoea*, *C. exaltata*, and *C. obtusiflora *vs.*Panax *across most genes present in *Epifagus***. Most genes are less altered relative to the outgroup in *Epifagus *than in *Cuscuta obtusiflora*, and the non-transcriptional/translational genes remaining in *Epifagus *(*clpP *and *accD*) are particularly divergent in *C. obtusiflora*.

**Figure 8:**
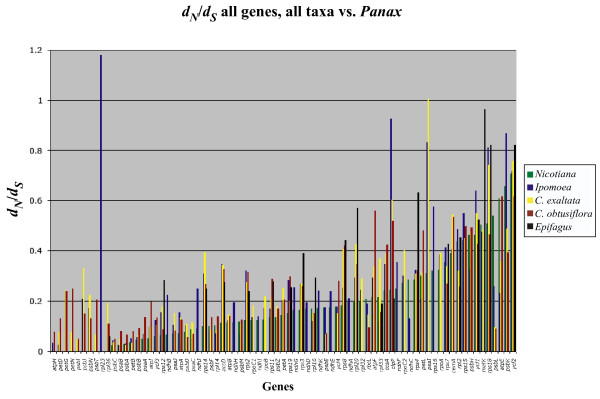
***d*_*N*_/*d*_*S *_for all genes, all taxa (including *Epifagus*) vs.*Panax***. Most genes evolve more quickly in *Ipomoea *than in *Nicotiana *(tobacco), indicating relaxed constraint on plastid genes even before evolution of parasitism in Convolvulaceae. Constraint is further relaxed in *Cuscuta exaltata *and is most relaxed in *Cuscuta obtusiflora*, although photosynthetically related genes remain highly constrained. In general, genes present in *Epifagus virginiana *are under higher levels of constraint than in *Cuscuta obtusiflora*, despite the retention of photosynthetic genes in *Cuscuta*.

We also wanted to test whether genes remaining in the plastid of the fully nonphotosynthetic *Epifagus *are under less constraint than those of the putatively photosynthetic *Cuscuta *species. Surprisingly, among the alignable genes they share, *C*. *obtusiflora *is usually more divergent at the protein level from a common outgroup, *Panax *(fig. 7). Comparison of *d*_*N*_/*d*_*S *_across all genes shows no clear trend, with some genes under greater constraint in *Epifagus *than in *C. obtusiflora *and others more conserved in *Cuscuta *(fig. 8).

*C. obtusiflora *retains the four protein-coding genes in *Epifagus *not related to transcription or translation and presumably the reason for retaining a plastid genome in that species: *accD*, *clpP*, *ycf1*, and *ycf2 *[8]. *accD *and *clpP *both are less constrained in *Cuscuta *than in *Epifagus*, and in *clpP*, dramatically so, with all three Convolvulaceae taxa exhibiting higher *d*_*N*_/*d*_*S *_for both genes. The effect this has on the amino acid divergence is also very apparent (fig. 7). *clpP *is a protease that is essential for shoot development in *Nicotiana*, but exactly which proteins it targets for degradation are still unknown [45]. Why it is so divergent in the closely related autotroph, *Ipomoea*, also has yet to be deduced. While alignable regions of *ycf1 *and *ycf2 *actually have lower *d*_*N*_/*d*_*S *_in *C. obtusiflora *than for *Epifagus*, other regions of each gene are unalignable at even the protein level in *Cuscuta *while *Epifagus *is relatively easy to align, and overall protein divergence is actually much higher for *C. obtusiflora *than *Epifagus *in these genes. Overall, with the exception of photosynthetic genes, the plastid genome of *Cuscuta obtusiflora *is more streamlined, less constrained, and more divergent than *Epifagus *for the genes they share in common. Whether this indicates faster overall evolutionary rates in *C. obtusiflora *or simply a longer time as a specialized parasite under relaxed constraint is difficult to discern without accurate dating methods and more taxon sampling.

### Comparisons with other Nonphotosynthetic Lineages

Despite some differences in patterns of evolution, many parallels exist between plastid genome evolution in *Cuscuta *and that of the related but independently derived parasitic lineage Orobanchaceae, including *Epifagus*. Both lineages show overall increased rates of nucleotide substitution, relaxed selective constraint, and lack any appreciable shift in synonymous codon usage in spite of loss of multiple tRNAs [46](Table 5). Substantial gene loss is observed in both lineages; in addition to sharing loss of all *ndh *and *rpo *genes with *C. obtusiflora*, *Epifagus *has lost a largely overlapping set of tRNAs from its plastid genome. All tRNAs lost in *C*. *obtusiflora *are also lost in *Epifagus *with the exception of *trnR*-ACG, and even that has been suggested to be a pseudogene [13]. The three ribosomal proteins lost in *C. obtusiflora *are also a subset of the six lost in *Epifagus*. Although *Epifagus *lacks all photosynthetic genes, other Orobanchaceae retain genes normally required for photosynthesis in seemingly functional form. *Lathraea clandestina *has what appears to be a functional *rbcL *(RuBisCo, large subunit) gene, and *rpo *genes are also amplifiable by PCR, despite the fact that the plant apparently lacks chlorophyll and spends its entire life cycle underground except when flowering [12]. Similarly, some members of the genus *Orobanche *and other holoparasites within the family retain *rbcL *genes that appear to be evolving under functional constraint [47-49]. *Pholisma*, a genus in the holoparasitic family Lennoaceae, is yet another example of an independently nonphotosynthetic lineage retaining *rbcL *[50]. Without full plastid genome sequence from these plants, it is difficult to know whether they too may still possess a necessary complement of plastid genes for residual photosynthesis, although unlike the *Cuscuta *species in this study, they lack obvious chlorophyll at any life stage and are not above ground to encounter light for most of their life cycle.

**Table 5:** Relative synonymous codon usage (RSCU) patterns across the plastid genomes

AA	Codon	N	RSCU	AA	Codon	N	RSCU
A. *Ipomoea *Cumulative Codon Usage							
Phe	UUU	907	1.33	Ser	UCU	463	1.53
	UUC	455	0.67		UCC	291	0.96
Leu	UUA	742	1.91		UCA	344	1.14
	UUG	467	1.2		UCG	184	0.61
Tyr	UAU	649	1.6	Cys	UGU	211	1.45
	UAC	160	0.4		UGC	80	0.55
ter	UAA		0	ter	UGA		0
ter	UAG		0	Trp	UGG	407	1
Leu	CUU	523	1.35	Pro	CCU	347	1.49
	CUC	149	0.38		CCC	194	0.84
	CUA	314	0.81		CCA	257	1.11
	CUG	135	0.35		CCG	131	0.56
His	CAU	392	1.49	Arg	CGU	284	1.23
	CAC	135	0.51		CGC	107	0.46
Gln	CAA	631	1.56		CGA	315	1.37
	CAG	180	0.44		CGG	112	0.49
Ile	AUU	957	1.5	Thr	ACU	505	1.61
	AUC	389	0.61		ACC	257	0.82
	AUA	562	0.88		ACA	358	1.14
Met	AUG	511	1		ACG	132	0.42
Asn	AAU	828	1.54	Ser	AGU	377	1.25
	AAC	247	0.46		AGC	155	0.51
Lys	AAA	938	1.49	Arg	AGA	410	1.78
	AAG	317	0.51		AGG	153	0.66
Val	GUU	437	1.45	Ala	GCU	591	1.83
	GUC	148	0.49		GCC	197	0.61
	GUA	448	1.49		GCA	353	1.09
	GUG	170	0.57		GCG	152	0.47
Asp	GAU	698	1.58	Gly	GGU	508	1.3
	GAC	186	0.42		GGC	171	0.44
Glu	GAA	900	1.47		GGA	579	1.48
	GAG	326	0.53		GGG	304	0.78
B. *C. exaltata *Cumulative Codon Usage							
Phe	UUU	769	1.41	Ser	UCU	355	1.55
	UUC	321	0.59		UCC	214	0.94
Leu	UUA	627	1.9		UCA	260	1.14
	UUG	424	1.29		UCG	149	0.65
Tyr	UAU	517	1.62	Cys	UGU	150	1.46
	UAC	123	0.38		UGC	55	0.54
ter	UAA		0	ter	UGA		0
ter	UAG		0	Trp	UGG	322	1
Leu	CUU	405	1.23	Pro	CCU	263	1.33
	CUC	130	0.39		CCC	187	0.95
	CUA	264	0.8		CCA	201	1.02
	CUG	128	0.39		CCG	139	0.7
His	CAU	350	1.48	Arg	CGU	242	1.22
	CAC	122	0.52		CGC	102	0.51
Gln	CAA	530	1.52		CGA	273	1.37
	CAG	168	0.48		CGG	117	0.59
Ile	AUU	753	1.5	Thr	ACU	428	1.67
	AUC	271	0.54		ACC	210	0.82
	AUA	482	0.96		ACA	280	1.09
Met	AUG	385	1		ACG	105	0.41
Asn	AAU	684	1.5	Ser	AGU	299	1.31
	AAC	226	0.5		AGC	93	0.41
Lys	AAA	860	1.56	Arg	AGA	326	1.64
	AAG	243	0.44		AGG	135	0.68
Val	GUU	372	1.42	Ala	GCU	464	1.73
	GUC	132	0.5		GCC	186	0.69
	GUA	378	1.44		GCA	292	1.09
	GUG	165	0.63		GCG	133	0.49
Asp	GAU	571	1.55	Gly	GGU	395	1.2
	GAC	167	0.45		GGC	173	0.53
Glu	GAA	716	1.48		GGA	462	1.4
	GAG	254	0.52		GGG	288	0.87
C. *C. obtusiflora *Cumulative Codon Usage							
Phe	UUU	649	1.51	Ser	UCU	262	1.56
	UUC	213	0.49		UCC	138	0.82
Leu	UUA	559	2.14		UCA	203	1.21
	UUG	292	1.12		UCG	108	0.64
Tyr	UAU	392	1.71	Cys	UGU	112	1.53
	UAC	67	0.29		UGC	34	0.47
ter	UAA		0	ter	UGA		0
ter	UAG		0	Trp	UGG	264	1
Leu	CUU	323	1.24	Pro	CCU	207	1.41
	CUC	88	0.34		CCC	109	0.74
	CUA	215	0.82		CCA	185	1.26
	CUG	92	0.35		CCG	88	0.6
His	CAU	245	1.54	Arg	CGU	188	1.39
	CAC	73	0.46		CGC	80	0.59
Gln	CAA	425	1.51		CGA	202	1.49
	CAG	138	0.49		CGG	62	0.46
Ile	AUU	593	1.59	Thr	ACU	358	1.85
	AUC	189	0.51		ACC	121	0.62
	AUA	339	0.91		ACA	208	1.07
Met	AUG	310	1		ACG	88	0.45
Asn	AAU	543	1.53	Ser	AGU	240	1.43
	AAC	167	0.47		AGC	57	0.34
Lys	AAA	697	1.58	Arg	AGA	207	1.53
	AAG	188	0.42		AGG	74	0.55
Val	GUU	282	1.46	Ala	GCU	348	1.61
	GUC	137	0.71		GCC	151	0.7
	GUA	235	1.22		GCA	253	1.17
	GUG	119	0.62		GCG	114	0.53
Asp	GAU	399	1.53	Gly	GGU	338	1.4
	GAC	123	0.47		GGC	126	0.52
Glu	GAA	548	1.48		GGA	345	1.42
	GAG	194	0.52		GGG	160	0.66

Calculations were made from all coding regions combined. Due to difference in genome content, the total number of codons was different for all taxa.

Other non-angiosperm plastid-containing parasite lineages also show patterns of plastid gene loss and loss of selective constraint. Apicomplexan apicoplast genomes, which are thought to be derived from a plastid ancestor, are more similar to *Epifagus *than *Cuscuta *in that they contain only a few genes not involved in transcription or translation, none of which are related to photosynthesis. However, none of the remaining non-transcriptional/translational genes are shared with *Epifagus*, and RNA polymerase genes are retained in apicoplast genomes [51]. The euglenoid parasite *Astasia longa *also has a highly reduced plastid genome but like *Cuscuta *retains *rbcL *[52]. Nonphotosynthetic algae are variable in plastid genome content; the only genes not involved in transcription or translation that are normally found in angiosperms in the plastid genome of *Helicosporidium *are *ycf1 *and *accD*, two of the four such genes retained in parallel by *Epifagus*[53]. *Prototheca *also retains ATP synthase genes, part of the photosynthetic apparatus also retained by *Cuscuta *but lost in *Epifagus*[54]. Although some parallels exist, each independently derived parasite lineage appears to follow a unique pathway in plastid genome reduction.

### Lipid Biosynthesis as a Hypothesis for Photosynthetic Gene Retention in *Cuscuta*

While it has been hypothesized that the plastid genome must be retained at least minimally in nonphotosynthetic organisms for the transcription of *trnE*, an essential product for tetrapyrrole synthesis in the plastid[55], retention of a conserved photosynthetic apparatus in *Cuscuta *suggests another important role for the plastid genome in these parasites. Because no atmospheric gas exchange occurs with chlorophyllous cells in *C. reflexa*, recycling of respiratory carbon dioxide has been presented as a hypothesis for retention of photosynthesis in that species, and although their source carbohydrates all apparently originate from the host, a net decline in carbon dioxide release is indeed detected in the presence of light [22]. However, recycling carbon dioxide back to carbohydrate through the Calvin cycle is not the only potential reason for retaining photosynthesis. Another possible explanation for conservation of photosynthetic genes in *Cuscuta *and retention of *rbcL *in other holoparasities may lie in a recently described alternative function of RuBisCo involving lipid biosynthesis, where it acts independently of its formerly known role in Calvin cycle production of carbohydrates. In this alternative pathway, 20% more acetyl-CoA is available for fatty acid biosynthesis, and 40% less carbon is lost as carbon dioxide in green seeds of *Brassica napus*. This pathway is still largely reliant on ATP and NADPH generated during the light reactions of photosynthesis, although less than 15% of that necessary for the Calvin cycle is needed for this function of RuBisCo to play a dominant role in lipid synthesis [56]. No atmospheric carbon dioxide would be necessary for this process, and it could also explain the observation of less respiratory carbon dioxide loss during light exposure [22], when necessary ATP and NADPH for the reaction would be produced. Chlorophyll is most concentrated in developing ovules and seeds of *Cuscuta obtusiflora *and close relatives in subgenus *Grammica *like *Cuscuta pentagona*, which has been shown to lack the circular ring of chlorophyllous cells between the pith and cortex [57]. Because *Cuscuta *species must survive long enough after germination to search for and attach to a host, utilizing this alternative function of photosynthesis for efficient lipid allocation to seeds and subsequent efficient carbon use in the free-living seedlings may explain the need for an intact photosynthetic apparatus in this parasitic lineage.

Use of RuBisCo for lipid biosynthesis may also explain retention of *rbcL *in other holoparasite angiosperm plastids and in the parasitic euglenoid *Astasia*. One of the few genes for which the plastid genome is transcribed in *Epifagus *and *Helicosporidium*, *accD*, is a subunit of acetyl co-A carboxylase, indicating that lipid biosynthesis remains an important function of plastids in these species as well. Lipid biosynthesis has already been experimentally demonstrated as a major function of apicoplasts and plastids in a number of nonphotosynthetic non-angiosperm species[58,59]. Future physiological study of photosynthetic tissues in *Cuscuta *as well as other parasitic plants, which may have largely if not entirely lost the primary photosynthetic function of their plastid genomes, should lead to greater understanding of possible alternate roles of the plastid genome in parasitic and autotrophic plants alike.

## Conclusion

By sequencing the full plastid genomes of two parasitic angiosperms in the genus *Cuscuta *and a non-parasitic close relative, *Ipomoea*, we have been able to gain a better understanding of the directional downsizing of the plastid genome in heterotrophs as dependence on photosynthesis for carbohydrate production decreases. These genomes are greatly complemented by comparison to that already existing for the parasitic angiosperm *Epifagus virginiana*; by studying the similarities and the differences between these two parasitic lineages, we have unveiled a clearer picture of which changes to the plastid genome might be expected to occur in all transitions to heterotrophy, which might be peculiar to those plants lacking chlorophyll, and which may be lineage specific to *Cuscuta*. We find *ndh *genes to be the only genes functionally lost during the transition to parasitism in *Cuscuta*, while more extensive loss of both coding and noncoding material has occurred more recently. Despite substantial reduction of the plastid genome in *Cuscuta*, the most highly conserved genes continue to be those directly involved in photosynthesis, whereas those genes shared with the fully nonphotosynthetic *Epifagus *are amongst the least conserved. We postulate that the extremely high levels of constraint on remaining photosynthetic genes may indicate an important function for these gene products in lipid biosynthesis rather than carbohydrate production through the Calvin Cycle.

## Methods

### Plastid Genome Sequencing, Assembly, and Annotation

Seeds of all three species were germinated and grown in the Pennsylvania State University Biology Greenhouse. An heirloom cultivar of *Ipomoea purpurea*, "Grandpa Ott's", was used to decrease likelihood of heteroplasmy within the sample. One gram of young leaf tissue was used for DNA isolation in *Ipomoea*. One gram of tissue from a collection of very green seedlings originating from a selfed parental plant was used for *Cuscuta exaltata*, and one gram of stem tip tissue was used for *Cuscuta obtusiflora*. Partial fosmid libraries were constructed from the extracted DNA using the CopyControl Fosmid Library Production Kit (Epicentre, Madison, WI). Libraries were screened for clones containing plastid DNA according to McNeal et al[60]. A subset of clones covering the entire plastid genome was selected for each species, and clones were shotgun sequenced and the reads assembled according to previously described methods [61]. Genome annotations were completed using DOGMA [62] in combination with manual sequence alignments of previously annotated genes from available related species. Sequences were deposited in Genbank with accession numbers EU118126 (*Ipomoea*), EU189132 (*C. exaltata*), and EU189133 (*C. obtusiflora*).

### Molecular Evolutionary Analyses

Phylogenies for each gene were constructed in PAUP*4.0b10 [63] under various Maximum Parsimony, Neighbor-Joining, and Maximum Likelihood criteria, including the following related taxa with full plastid genome sequences publicly available: *Nicotiana tabacum *(Genbank accession NC 001879) and *Atropa belladona *(NC 004561)(Solanaceae, Solanales, Asteridae), *Panax ginseng *(NC 006290)(Araliaceae, Apiales, Asteridae), and *Spinacia oleracea *(NC 002202)(Caryophyllidae) as an outgroup. All sequenced genes appeared orthologous, with only minor, method-dependent aberrations from the expected phylogeny which were probably due to extreme rate heterogeneity between taxa [64]. Maximum Likelihood analyses that were performed under the General Time-Reversible model with gamma distribution of among-site variation (GTR+gamma) and model parameters estimated from the data were most accurate at obtaining the expected phylogeny from the data and were used for subsequent phylogenetic reconstruction of combined-gene datasets.

Pairwise estimates of synonymous substitutions per synonymous site (*d*_*S*_) and nonsynonymous nucleotide substitutions per nonsynonymous site (*d*_*N*_) and standard errors were computed under the Kumar method using MEGA 2.1 [65] for each gene and for classes of genes that together encode subunits of larger proteins. ATP synthase genes, 6 genes, 4344 aligned characters (*atp*); cytochrome b6/f complex subunits, 6 genes, 2622 aligned characters (*pet*); photosystem I and II protein subunits, 19 genes, 11730 aligned characters (*psa *and *psb *= *ps*); large and small ribosomal protein subunits, 17 genes, 7686 aligned characters (*rpl *and *rps = rp*); plastid-encoded RNA polymerase 4 genes, 11958 aligned characters (*rpo*); and NADH-dehydrogenase, 11 genes, 10,653 aligned characters (*ndh*) were predefined classes of genes examined. Pairwise *d*_*S*_, *d*_*N*_, and amino acid p-distance were also calculated between *Epifagus virginiana *and *Panax*, the closest available outgroup to both it and *Cuscuta*.

Maximum Likelihood estimates of synonymous substitution rates and the ratio of nonysnonymous to synonymous rates (R) for each branch of the combined-gene dataset phylogenies were calculated under the MG94 × HKY 3 × 4 codon model in the HYPHY .99beta package [66]. HYPHY was also used to conduct likelihood ratio tests (LRTs) between trees with universally constrained synonymous rates and R versus trees with each respective parameter free from constraint on one branch. Branches leading to Convolvulaceae (*Ipomoea *+ both *Cuscuta *species), *Cuscuta*, and each individual *Cuscuta *species were tested for significant p-values in this manner. Additionally, pairwise relative rates tests were conducted for each gene class using various combinations of taxa with all parameters constrained as the null hypothesis and all parameters unconstrained as the alternate hypothesis. Pairwise relative ratio tests were conducted in HYPHY between the combined datasets with either synonymous or nonsynonymous distances constrained as the null hypothesis to determine whether there was significant heterogeneity in either across gene classes. Parameters were estimated independently for each branch. Finally, GCUA [67] was used to determine relative synonymous codon usage across all coding sequences for each genome to identify any changes in codon bias that may have accompanied tRNA loss or relaxed selection for photosynthesis.

## Note in Proof

Two additional *Cuscuta *plastid genome sequences were published (Funk et al. *BMC Plant Biology *2007, **7**:45) during the late stages of review of our manuscript. Although the analyses of the data in our manuscripts demonstrate little overlap, the plastid genome structure and content of *Cuscuta reflexa *in that manuscript closely parallels our data from *Cuscuta exaltata*. Likewise, the sequence of *Cuscuta gronovii *demonstrates similar structure and gene content as *Cuscuta obtusiflora*.

## Competing interests

The author(s) declares that there are no competing interests.

## Authors' contributions

JRM cultivated the plants, produced and screened the partial genomic fosmid libraries, annotated and analyzed the plastid genomes, and drafted the manuscript. JVK subcloned, sequenced, and assembled reads for the selected fosmid clones. JLB and CWD helped conceive, design, and coordinate the study and made large contributions to manuscript editing. All authors read and approved the final manuscript.
